# Induced oscillatory brain responses under virtual reality conditions in the context of repetition priming

**DOI:** 10.1007/s00221-023-06766-8

**Published:** 2024-01-10

**Authors:** Joanna Kisker, Marike Johnsdorf, Merle Sagehorn, Benjamin Schöne, Thomas Gruber

**Affiliations:** 1 Institute of Psychology, Osnabrück University, Osnabrück, Germany; 2 Department of Psychology, Norwegian University of Science and Technology, Trondheim, Norway

**Keywords:** Induced oscillatory responses, Virtual reality, Gammaband response, Repetition priming, Repetition suppression, Electroencephalography

## Abstract

In the human electroencephalogram (EEG), induced oscillatory responses in various frequency bands are regarded as valuable indices to examine the neural mechanisms underlying human memory. While the advent of virtual reality (VR) drives the investigation of mnemonic processing under more lifelike settings, the joint application of VR and EEG methods is still in its infancy (e.g., due to technical limitations impeding the signal acquisition). The objective of the present EEG study was twofold. First, we examined whether the investigation of induced oscillations under VR conditions yields equivalent results compared to standard paradigms. Second, we aimed at obtaining further insights into basic memory-related brain mechanisms in VR. To these ends, we relied on a standard implicit memory design, namely repetition priming, for which the to-be-expected effects are well-documented for conventional studies. Congruently, we replicated a suppression of the evoked potential after stimulus onset. Regarding the induced responses, we observed a modulation of induced alphaband in response to a repeated stimulus. Importantly, our results revealed a repetition-related suppression of the high-frequency induced gammaband response (>30 Hz), indicating the sharpening of a cortical object representation fostering behavioral priming effects. Noteworthy, the analysis of the induced gammaband responses required a number of measures to minimize the influence of external and internal sources of artefacts (i.e., the electrical shielding of the technical equipment and the control for miniature eye movements). In conclusion, joint VR–EEG studies with a particular focus on induced oscillatory responses offer a promising advanced understanding of mnemonic processing under lifelike conditions.

**Supplementary Information:**

The online version contains supplementary material available at 10.1007/s00221-023-06766-8.

## Introduction

The recent striving for more lifelike settings in psychological research has frequently been implemented by translating classical research paradigms to virtual reality (VR) or advancing them within these immersive settings (e.g., de la Rosa and Breidt 2018; Kothgassner and Felnhofer 2020; Pan and Hamilton 2018; Parsons et al. 2020; Slater and Sanchez-Vives 2016; see also Nastase et al. 2020). Declared a great benefit, VR setups allow for similar perceptual features of the stimuli’s real-life equivalents, foremost the presentation of 3D objects within a surrounding and congruent environment (Peeters 2018; Tromp et al. 2020). While the number of studies taking advantage of these characteristics is increasing, the combination with electrophysiological examinations is markedly less widespread compared to conventional EEG-based approaches (e.g., Weber et al. 2021). To date, only a relatively small number of studies exist that provide evidence that the acquisition and analyses of lower frequency oscillations below 50 Hz are little restricted in high-quality EEG measurements per se (e.g., Cattan et al. 2018; Tauscher et al. 2019; Weber et al. 2021). Given this technical feasibility, the joint application of VR and EEG offers a promising approach for the examination of cognitive processes and mechanisms under lifelike settings, for example, of cognitive load (Dan and Reiner 2017), attentional (Schubring et al. 2020), and motivational processes (Schöne et al. 2021a) as well as mnemonic processes (Kisker et al. 2021) on the electrophysiological level.

In particular, the immersive features of VR offer contextual information to the experimental stimuli, which is essential for episodic memory (see Rugg and Curran 2007). Due to these contextual information and aforementioned sensory proximity to real environments, VR fosters the transfer and generalizability of experimental results to real-life processes (e.g., Kisker et al. 2021; Parsons 2015; Smith 2019; Smith and Mulligan 2021). Hence, studies combining VR and EEG have great potential for this field of research in particular. Most existing studies focusing on mnemonic processes under VR conditions examine event-related potentials (ERPs), e.g., during spatial learning tasks (Plank et al. 2013), the association of object-location relations (Plank et al. 2015), working memory (Aksoy et al. 2021) or priming effects (Johnsdorf et al. 2023). However, the analysis of induced oscillatory activity offers more differentiated insights into processes which are not precisely time-locked to stimulus onset (like implicit and explicit memory retrieval; see e.g., Jaiswal et al. 2010; Kisker et al. 2021; Klotzsche et al. 2023). Induced activity occurs with a jitter in latency from one trial to the next (Eckhorn et al. 1990) and it is typically extracted through frequency-domain averaging of single trials. Thus, the induced oscillatory signal contains information which would average out in the time-domain ERP.

On this background, we designed a joint VR–EEG study to examine induced oscillations reflecting mnemonic processing under VR conditions. We translated a classical repetition priming paradigm to VR conditions, presenting 3D objects repeatedly in a congruent environment. This specific paradigm is related to implicit memory processes (Gotts et al. 2012) and was chosen because the effect was replicated numerous times and its electrophysiological indices are well-documented (e.g., Auksztulewicz and Friston 2016; Grill-Spector et al. 2006; Gruber et al. 2004b; Snyder and Keil 2008). The paradigm is associated with the repetition suppression effect: upon the first presentation of an object, a large neural network fires in synchrony to process the visual input and to integrate it into a coherent object representation (Desimone 1996; Grill-Spector et al. 2006; Gruber et al. 2004; Wiggs and Martin 1998). Upon repeated presentation of the very same object, this network is proposed to sharpen, i.e., the neural processing of the presented objects gets more efficient since only a smaller portion of the same network fires to process the object. This sharpening mechanism results in the repetition suppression effect affecting both behavioral and electrophysiological outcomes. In particular, this priming effect is mirrored in behavioral responses linked to the stimulus, e.g., a required key press, which are carried out faster when a stimulus is presented a second time compared to the first time (i.e., has been primed; e.g., Gruber et al. 2004; Hassler et al. 2011). The neural counterpart of behavioral priming is reflected in a lower event-related potential (ERP) amplitude at posterior electrode sites in response to repeated compared to first presentations of stimuli (e.g., Gruber et al. 2004; Gruber and Müller 2002). While some well-known memory indices cannot be transferred from classical paradigms to VR paradigms without restriction, for example the theta old/new effect (Kisker et al. 2021), the repetition suppression effect is evident at the level of ERPs under VR conditions (Johnsdorf et al. 2023). Since the same effect can be observed under VR conditions and under conventional approaches, it might indicate that this effect can also be replicated with other associated measures, like induced oscillatory indices.

Beyond the ERP, repetition priming is reflected in modulations of both low- and high-frequency responses, offering the opportunity to evaluate a broad response under VR conditions. Since repetition priming is primarily associated with implicit memory processes (Gotts et al. 2012), the most frequently examined oscillatory responses are the induced gammaband response (iGBR) and the induced alphaband response (iABR). Most importantly, the iGBR synchronizes in response to simultaneously firing neurons encoding the same object as a neural correlate of the sharpening mechanism. Hence, the first presentation of an object is associated with a relatively higher iGBR compared to repeated presentation of the same object (e.g., Grill-Spector et al. 2006; Gruber et al. 2004b; Snyder and Keil 2008). Vice versa, the alphaband responds in the opposite way as the gammaband: it is inversely related to cortical activity and a decreased iABR is associated with increased cortical activity (Davidson et al. 2000; Harmon-Jones et al. 2010; Klimesch 1999; Neuper and Pfurtscheller 2001). Hence, initial stimulus presentation is accompanied by a more negative iABR compared to repeated presentations (Kim et al. 2020; Snyder and Keil 2008). In contrast to both aforementioned frequency bands, the induced thetaband response (iTBR) is more strongly related to explicit memory processes, like episodic memory retrieval (e.g., Gruber et al. 2008; Gruber and Müller 2006; Klimesch et al. 1997; Nyhus and Badre 2015). Some rare findings indicate spectral changes in the thetaband range in response to mnemonic processing during repetition priming paradigms as well, indicating the involvement of executive control even during repetition priming (Graetz et al. 2019). Even though priming is primarily attributed to implicit memory, it might still serve as a mechanism in the formation of long-term memories (Gotts et al. 2012), making the examination of the iTBR essential to complete the overall picture.

Yet the analysis of high-frequency oscillations has been largely omitted from studies implementing VR conditions. In particular, the induced gammaband response (iGBR, approx. 30–90 Hz) has numerous significant functions in human cognition. Its synchronization is associated with the integration of perceptual features in bottom-up processes driving object representation, as well as with attentional and mnemonic top-down processes (Köster and Gruber 2022). The maintenance of mental representations in working memory as well as successful encoding and retrieval of long-term memory relies on the gammaband’s synchronization (Jensen et al. 2007). Consequently, the high-frequency domain offers a crucial complement and extension of insights into cognitive processes beyond the lower frequency ranges. On the downside, higher frequency oscillations are more prone to electrical interference than low-frequency oscillations and approached with even more caution in joint VR settings.

In particular, most EEG systems were not originally designed for placing an additional electrical device, like a head-mounted display, on top of the sensors. The resulting mechanical pressure (e.g., Klug and Gramann 2021) and line hum from the electrical device might easily distort the likewise weak signals like the iGBR to be derived from the surface of the scalp (e.g., Hertweck et al. 2019; Weber et al. 2021). Previous studies examining processes associated with the iGBR under VR conditions worked around this issue by sticking to the lower gammaband range (<50 Hz) and, in some cases, by applying a bandpass filter cutting off the alternating current range or a notch filter correcting for it (50/60 Hz, respectively; e.g., Kim et al. 2001; Tarrant and Cope 2018; Wang et al. 2020). Others implemented less immersive, so-called desktop-VR settings, i.e., three-dimensionally created environments presented two-dimensionally on a screen to circumvent external interference (Calabrò et al. 2017; Goo et al. 2006; Vivekananda et al. 2021). However, these approaches set strict limitations on which subranges of the iGBR may be analyzed, and thereby categorically exclude the examination of modulations in the higher frequency range above 50 Hz under VR conditions.

The strongest interference in the frequency range above 50 Hz seems to originate from line hum, its harmonics, and the head-mounted displays-specific refreshing rate (Weber et al. 2021). Whether the head-mounted display’s refreshing rate induces artifacts seems to depend on the specific model: while the Oculus Rift did induce artifacts in the 90 Hz range, the HTC Vive did not (Weber et al. 2021). Moreover, aforementioned studies did not specify whether the experiments were conducted in an electrically shielded room, i.e., a faraday cage, as would be appropriate for EEG studies that aim to examine high-frequency oscillations (see e.g., Busch et al. 2004). Since some VR headsets run on direct current, e.g., the HTC Vive Pro and Pro2, the possibility that other electrical sources in the room are causing the electrical interference in the 50 Hz range, not the head-mounted display itself, could not be ruled out in rooms containing further electrical sources like devices and sockets. Consequently, the issue of interference by means of line hum might be solved by performing joint VR–EEG experiments in a faraday cage in which no device plugs, sockets or similar are located. If no line hum would occur under these circumstances while using a VR head-mounted display, this would suggest that wearing it is not necessarily problematic for the evaluation of the iGBR.

Yet the iGBR bears an additional artifact when examined by means of scalp-recorded EEG as it is correlated with the transient spike potentials generated by miniature saccades (MS, Yuval-Greenberg et al. 2008). It has, therefore, been questioned whether the typical peak in the iGBR around 200–300 ms after stimulus onset reflects synchronous neuronal oscillations associated with cognitive processes such as object representations, memory, and attention (Yuval-Greenberg et al. 2008). Corresponding to the 30–100 Hz range, these muscular artifacts superimpose iGBR modulations potentially driven by cognitive modulations (Fries et al. 2008a, b). Addressing this concern, Hassler and colleagues (Hassler et al. 2011) developed and validated an ICA-based algorithm ***co****rrecting for ****s****accade-related ****tra****nsient ****p****otentials* (COSTRAP). After application of COSTRAP, the remaining iGBR was sensitive to object recognition with its source being located in bilateral temporal areas, indicating cortical rather than muscular activity (Hassler et al. 2011).

Summing up, the objective of our study was twofold: our approach aimed to examine the feasibility of investigating induced oscillatory responses of different frequency ranges under VR conditions, i.e., whether the investigation of induced oscillations under VR conditions yield equivalent results compared to standard paradigms. We focused, in particular, on whether modulations of the high-frequency range occur equivalently to conventional EEG paradigms, while the consideration of the lower frequencies contributes to the integration into the existing research body. Second, we aimed at obtaining further insights into basic mnemonic processes under lifelike conditions. The results of the study are particularly relevant for subsequent studies in the field of mnemonic processing which include the analyses of high- and low-frequency oscillations to examine complementary processes, like familiarity and recollection of previously encoded stimuli (e.g., Gruber et al. 2008).

To check whether our paradigm is generally suitable to elicit the effect under consideration, the behavioral priming effect by means of response times as well as the EEG data at ERP level is examined as a first step. Both, a lower response time and a lower ERP amplitude in response to the repeated presentation of stimuli would indicate successful priming. Going one step further, potential modulations of the iABR, iGBR, and iTBR are examined (see Gruber et al. 2004 for a similar procedure). For the iABR, we expect a more negative response to initial presentations compared to repeated presentations measured at posterior sensors (Kim et al. 2020; Snyder and Keil 2008). Moreover, we hypothesize to replicate the repetition suppression effect by means of a difference between the iGBR to initial and to repeated presentation of objects in VR, i.e., a relatively higher iGBR for first presentations given that the head-mounted display does not induce electrical interference in the 50 Hz range and its harmonics. Based on previous studies, this effect is expected to set in no earlier than 200 ms after stimulus onset and to be maximal at posterior electrodes (see e.g., Hassler et al. 2011). Albeit the heterogenous results concerning the iTBR in repetition priming paradigms (see e.g., Graetz et al. 2019; Gruber et al. 2004), we assume that the iTBR will not differentiate between first and repeated presentations in this repetition priming paradigm due to its primary association with explicit memory processes (see e.g., Gruber et al. 2004).

## Methods

### Participants

The study was conducted in accordance with the declaration of Helsinki and was approved by the ethics committee of Osnabrück University. All participants gave informed consent and were blind to the research question. They received either partial course credits or 15€ for participation. A required sample size of 26 was determined using G*Power (Faul et al. 2007). Since the iGBR is the oscillatory response that is most likely to be limitedly accessible in the joint VR–EEG setting, the effect size *f* was estimated from the repetition suppression effect in the iGBR of previous studies with *f* = 0.3 (Gruber et al. 2004). Since previous studies analyzed the repetition suppression effect by means of rmANOVAs, the power analyses were based on the rmANOVA parameters even though, when appropriate, within-group comparisons were performed as paired *t *tests and rmANOVA was only used when more than one factor was included in the analyses, i.e., for the thetaband response. The determined sample size is almost twice as large as in conventional repetition suppression studies (e.g., Friese et al. 2012a, b; Gruber et al. 2004; Gruber and Müller 2002) but comparable to previous VR–EEG studies (e.g., Johnsdorf et al. 2023; Lange and Osinsky 2020). To cope with potential technical issues during EEG acquisition, 32 participants were recruited from the student population of Osnabrueck University. An anamnesis was obtained from all participants by means of a short interview by the principal investigator. Those participants who reported suffering from psychological or neurological disorders were excluded from participation. All participants had normal or corrected-to-normal sight. When vision correction was necessary, only those participants who had contact lenses could participate, not those who wore glasses. The first two datasets were used to pilot the technical setup and were, therefore, not included in the analyses. One participant was excluded from analyses due to pronounced external electrical interference (see Electrophysiological recordings and preprocessing). The final sample for data analyses comprised 29 data sets (*M*_age_ = 23.00; SD_age_ = 3.04; 2 left-handed, gender: 22 female, 7 male, none diverse; sex and gender were equal for all participants). Twenty-seven participants had prior experience with VR head-mounted displays but none used them on a regular basis.

### Stimulus material

The stimuli used were 3D objects from a validated database (Peeters 2018). The database includes 147 objects of which 140 were used. The remaining objects that were not used either lacked texture and color (three objects) or were too flat to be clearly visible within the virtual environment (four objects, e.g., scattered papers and newspaper). Of the 140 objects used, 33 depicted edible objects and were used for the behavioral task (see below). We aimed for a proportion of approximately 20% of all trials requiring a response, as this proportion has been sufficient in previous studies on repetition priming (Gruber et al. 2004). Dividing the objects into edible and non-edible objects yielded 23.5% for edible objects, which was the best approximation to 20% possible with the given validated stimulus set. All trials that required a motoric response were used for behavioral analyses, but excluded from electrophysiological analyses to prevent motoric artifacts. Accordingly, 107 trials were available for EEG analyses per condition (first presentation, second presentation). In accordance with conventional repetition priming paradigms, each stimulus was presented twice concerning the experimental trials (e.g., Gruber and Müller 2002). To prevent habituation or expectancy effects beyond priming, the second presentation followed the first stimulus presentation either directly (lag 0), with one other stimulus in between (lag 1) or four other stimuli in between (lag 4; 33.33% each, see e.g., Gruber et al. 2004b; Gruber and Müller 2002 for a similar procedure). Five further stimuli were used from the OpenVirtualObjects database (Tromp et al. 2020) and only used for the training trials. The OpenVirtualObjects database has a comparable visual quality as the database used for experimental trials (Peeters 2018). The stimuli chosen for the training trials did not semantically overlap with the experimental trials. The decision to use the former database (Peeters 2018) for the experimental trials was based on it providing a larger number of objects. The stimuli used for the training trials were only presented once, as the participants task was not dependent on the repetition of the stimuli. Hence, the training aimed to familiarize participants with the overall procedure of the experiment and to clarify in which cases they were to press the game pad button.

### Procedure

For the test procedure, participants were seated in an electrically shielded cabin (faraday cage), equipped with a mobile EEG (see electrophysiological recordings) and an HTC Vive Pro 2 (HTC, Taoyuan, Taiwan). The HTC Vive Pro 2 offers 5 k resolution and a frame rate of 120 Hz. Any device outlets and computers were placed outside the shielded cabin. The exception was the EEG system and the HTC Vive Pro 2 including two base stations, with any power feeds installed outside the cabin via a channel sealed with steel wool.

The virtual environment was created using Unity 5 (Unity Technologies, San Francisco, United States). Within the virtual environment, participants were seated at a table with a distance of 90 cm to the position at which the stimuli would be presented. They were instructed to press a button on a conventional gamepad whenever the stimulus they saw depicted something edible. In any other case, no response was required. It was counterbalanced across participants whether they needed to press a button with their left or right index finger.

**Fig. 1:**
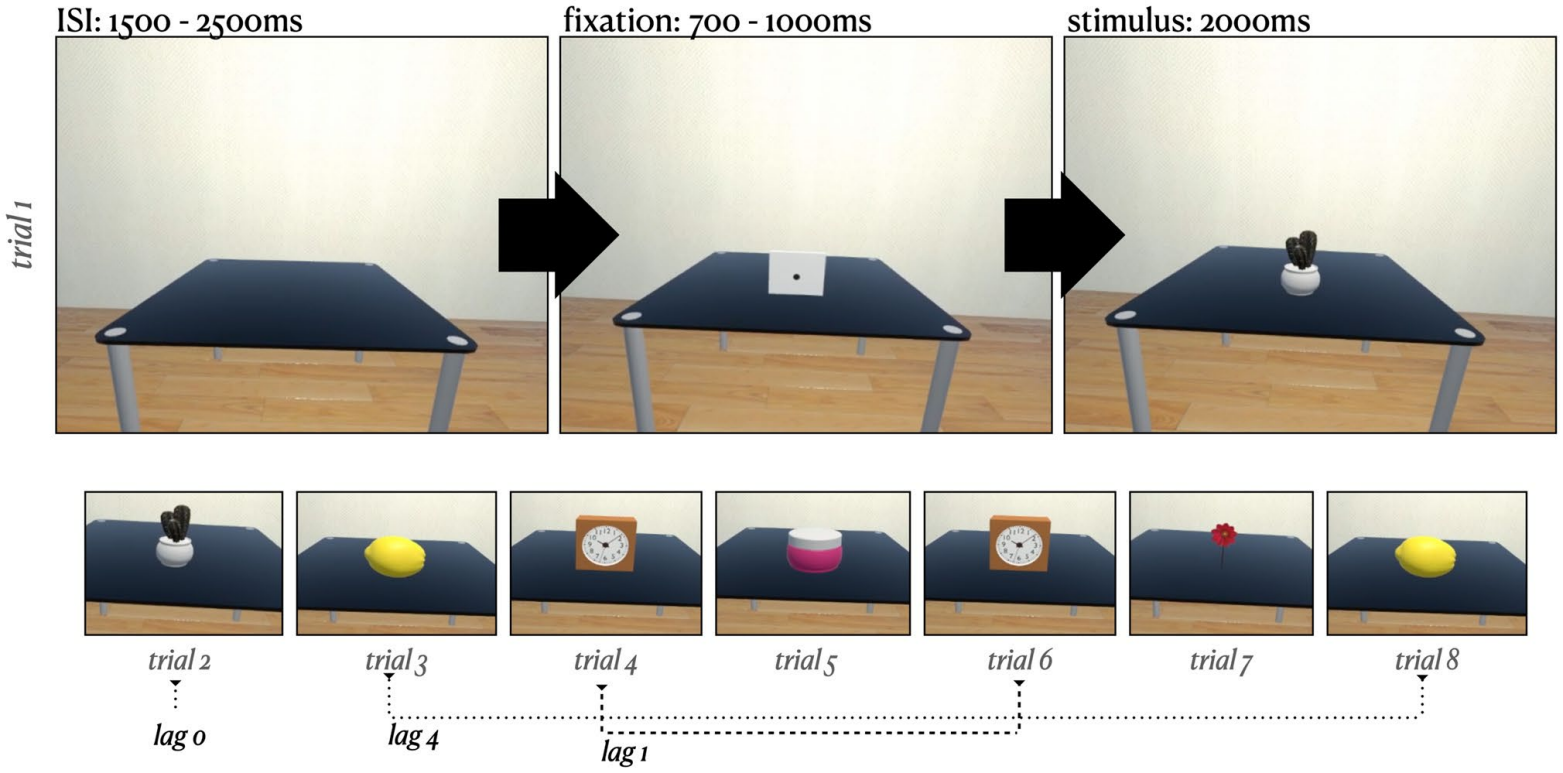
Visualization of the experimental trials. *Note*: each trial started with a clear table followed by a fixation phase. The fixation was realized as a cube that covered the full presentation area and was centered in the center of gravity for all objects. After the offset of the fixation, an object was presented for 2000 ms as the stimulus. The depicted stimuli are from the OpenVirtualObjects database (Tromp et al. 2020). *ISI* interstimulus interval

The experiment was conducted as a within-subject design and consisted of 5 training trials and 280 experimental trials. The start of the training trials, the start of the experimental trials, and the end of the break were indicated by a cube with a start symbol. Vice versa, the end of the training trials, the beginning of the break, and the end of the experiment were indicated by a cube with a pause symbol. Each trial consisted of a fixation (700–1000 ms), the presentation of a 3D object (2000 ms) and an interstimulus interval (1500–2500 ms, see Fig. 1). After the training trials, participants were given the opportunity to clarify any uncertainties about the task. After 140 experimental trials, a 3-min break was automatically made to prevent fatigue and muscular tension. During this break, the VR simulation was not interrupted. Participants remained seated within the same virtual environment, looking at the empty table.

All stimuli were presented on the table within a fixed presentation area of 20 cm^3^. Each stimulus was maximized within this area, i.e., depending on the ratio of the object, each stimulus was either 20 cm in height or 20 cm in width and presented centered within the presentation area. The fixation was implemented by a white cube which covered the presentation area in height and width. It had a centered black dot on its front which was positioned in the center of gravity of all stimuli. The stimulus size and the participants’ seating distance resulted in a viewing angle of 12.68° × 12.68°. To accurately determine the viewing angle, the actual distance between each current stimulus and the VR headset was determined per trial. This allowed for obtaining the individual mean viewing angle per participant, from which the mean viewing angle of 12.24° × 12.24° was calculated for the total sample (SD = 0.40°; range = 11.46°, 13.18°]).

### Behavioral data

To examine the priming effect on the behavioral level, the response time to stimuli depicting edible objects was assessed (time between stimulus onset and motoric response; 23.6% of all trials). These trials were not included in the EEG analyses. The mean response time was calculated as the time from stimulus onset to the participants’ response separately for initial and repeated presentations.

### Electrophysiological recordings and preprocessing

An electroencephalogram (EEG) with 64 active electrodes was recorded during the VR paradigm. The mobile EEG system LiveAmp64 by Brain Products (Gilching, Germany) was used. The electrodes were applied in accordance with the international 10–20 system and the electrode FCz served as an online reference, while AFz was used as the ground electrode. An online bandpass filter of 0.016–250 Hz was applied during the recording of the data. To achieve a good signal-to-noise ratio, the impedance of all electrodes was kept below 10 kΩ. For two participants, it was not possible to reduce the impedance below this limit, so all electrodes of these two participants were kept below 15 kΩ. In addition, the live signal was checked for line noise and slow drifts and corrected before starting the paradigm. The data were recorded at a sampling rate of 500 Hz. Triggers marking the onset of each stimulus and the participants responses were transmitted from Unity using Lab Streaming Layer (by SCCN; https://github.com/sccn/labstreaminglayer), which was used to synchronize the EEG data stream and Unity event triggers. The accuracy of the timing of the event triggers was cross-checked and corrected using a photodiode.

The EEG data were further preprocessed using MATLAB (version R2022a, MathWorks Inc) and EEGLAB (version 2023, Delorme and Makeig 2004). To exclude potential interference from external electrical signal prior to analysis, a fast Fourier transformation (FFT) was calculated separately for each raw data set. Prior to the calculation of the FFT, the raw dataset was re-referenced to average reference, i.e., the average of all electrode sides was subtracted from each separate electrode. The data were epoched around the stimulus onset [−500 to 1500 ms] and baseline corrected from 500 ms before to stimulus onset. The FFT was calculated separately per trial. For each participant, the FFT was visually checked for peaks in the 50 Hz domain and their harmonic frequencies across all trials (see Luck 2014, p. 220f). One participant was excluded due to excessive peaks in the 50 and 100 Hz domains (see Methods: Participants): for this participant, the mean power of 50 Hz was 12 SD higher than the mean of the average of 39–49 Hz, which was consequently considered to be line noise. The peaks were most likely caused by a power bank left within the shielded cabin (see Discussion).

For the analysis of the ERP, the raw continuous data were re-referenced to average reference. A high-pass filter of 1 Hz and a low-pass filter of 30 Hz were applied. The relatively high high-pass filter was chosen due to recommendations for mobile EEG setups with the intention to eliminate slow drifts which may occur, e.g., due to mechanical pressure (Klug and Gramann 2021). To remove artifacts, the EEGLAB function *clean raw data and artifact subspace reconstruction* (ASR; Kothe and Makeig 2013) was used with the default parameters for channel removal, but the burst correction/rejection and the additional removal of data periods disabled. Bad channels identified by ASR were interpolated. On average, less than one channel per participant was rejected and subsequently interpolated (*M* = 0.97; SD = 1.18). None of the electrodes used for analysis of the ERP was rejected or interpolated. Afterward, each channel was detrended separately. An independent component analysis was applied to identify and remove artifacts from eye movements, muscle or cardiac electrical activity (probability threshold 90% each). No subsequent measures of artifacts were regarded after artifact correction. The continuous data were resampled to 512 Hz and segmented into epochs from −500 to 1500 ms after stimulus onset. The data were baseline corrected from −400 to −100 ms before stimulus onset. Trials which required a motoric response from the participants (i.e., edible objects) were excluded. The ERP was computed as the average across trials per condition (first presentation (FP), second presentation (SP)). For analyses, posterior electrodes (POz, PO3, PO4, Oz, O1, O2) and a time window from 450 to 650 ms after stimulus onset were chosen based on previous studies (see Gruber et al. 2004; Gruber and Müller 2002).

For the analyses of the frequency domain, the raw continuous data were resampled to 512 Hz and segmented into epochs from −500 to 1500 ms after stimulus onset. Each channel was detrended separately. A baseline correction from −400 to −100 ms before stimulus onset (Gruber et al. 2004) was conducted before filtering (low pass 3 Hz, high pass 100 Hz) and re-referencing to average reference. Trials containing severe blinks were excluded by means of *statistical control of artifacts in dense array studies* (SCADS, Junghöfer et al. 2000). The frontal electrodes FP1 and FP2 were used for ocular artifact detection and rejection. On average, 6.79 (SD = 10.18) trials were rejected due to blinks. Subsequently, an algorithm was used for the correction of saccade-related transient potentials (COSTRAP, Hassler et al. 2011). In short, COSTRAP is an algorithm based upon independent component analysis (ICA) which detects artifacts caused by miniature saccades (MS, see Introduction) and removes them from the data. The full procedure is described in detail elsewhere (Hassler et al. 2011). For the detection of the MS-related artifacts, the frontal electrodes FP1 and FP2 were used. For the first run of COSTRAP, the time window for artifact correction was limited to 100–400 ms after stimulus onset, during which MS tend to cluster (e.g., Yuval-Greenberg et al. 2008). In a second run, the entire epoch was included to cope for residual artifacts beyond the typical cluster. Per individual dataset, four MS-related components were identified and removed. After COSTRAP application, flatline identification was applied, but no flatliners were detected and hence, no channels were interpolated. Remaining components which were with at least 90% probability identified as a muscle artifact, with at least 80% probability as an eye movement artifact, and with at least 95% probability line noise or channel noise, were removed by an ICA using eeglab. Trials requiring a motoric response were excluded from analyses and the grand mean was calculated separately for FP and SP. Congruent with the artifact correction for the ERP analyses, no subsequent measures of artifacts were regarded after artifact correction.

The spectral changes in oscillatory activity were analyzed by means of a Morlet wavelet analysis with a width of seven cycles per wavelet which is recommended for analyses of the gammaband range (see e.g., Gruber et al. 2004, 2008; Tallon-Baudry and Bertrand 1999). 200 wavelets from 1 to 100 Hz were calculated with a frequency resolution of 0.5. The procedure allows for a time-by-frequency (TF) representation of the data by providing a time-varying magnitude of the signal per frequency band. In general, the induced oscillatory activity tends to cancel out in the averaged evoked potential due to a jitter in its latency. To avoid canceling out the signal of interest (i.e., the induced response), the TF amplitude was averaged across single-trial frequency transformations. This procedure allows for analyzing non-phase-locked components. Moreover, the evoked response (i.e., the ERP) was subtracted from each trial before conducting the frequency decomposition as we focused on analyzing the non-phase-locked components of the signal (for details see e.g., Busch et al. 2006).

**Fig. 2:**
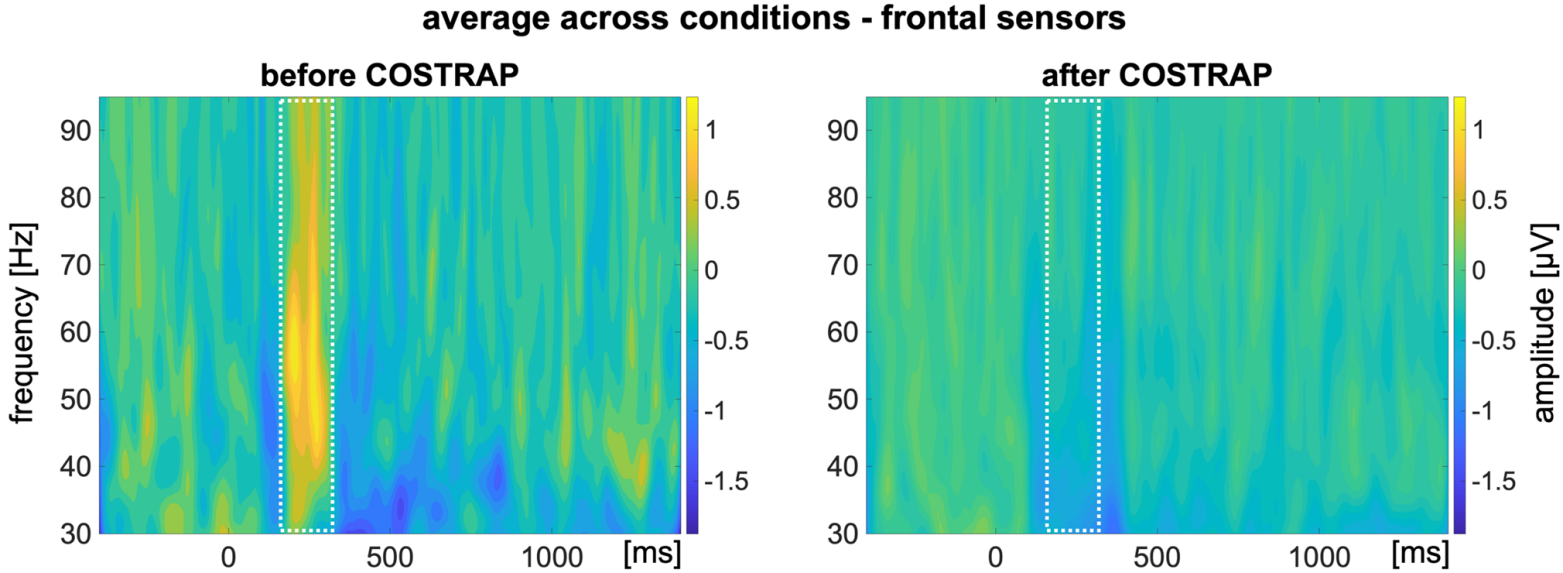
Time–frequency plot of the 30–95 Hz range for frontal electrodes. *Note:* time–frequency plot of the 30–95 Hz range for frontal electrodes equivalent to FP1 and FP2 before and after applying COSTRAP to the data. While the TF plot shows a strong increase in the gammaband range in the typical time window of miniature saccades before application of COSTRAP (*left*), this response is strongly reduced after application of COSTRAP (*right*), indicating successful minimization of the artifact

To check for successful minimization of the MS-related artifact, the data were first visualized for the frontal electrodes (FP1, FP2) in a TF plot and visually counterchecked against the TF plot of the data before applying COSTRAP. The TF plot in Fig. 2 (left panel) shows a pronounced increase in the iGBR power from approximately 180–250 ms which is indicative of MS-related transient potentials. After applying COSTRAP, there is no visible increase, indicating successful minimization of the artifact (see Fig. 2, right panel)

The electrodes and time windows chosen for further analyses were derived from previous repetition priming paradigms (Gruber et al. 2004b; Gruber and Müller 2002; Hassler et al. 2011). The specific frequency ranges and time windows were adapted by means of visual inspection of the TF plots. Per frequency, the region of interest was determined based on previous memory studies (see e.g., Hsieh and Ranganath 2014; Nyhus and Curran 2010; Gruber et al. 2004; Hassler et al. 2011). The central electrode of the respective region was mapped in a TF plot of the difference between first and second presentations: Pz for the iGBR, POz for the iABR, and Fz for the iTBR (see Results). Moreover, the electrodes derived from previous literature were counterchecked and adapted by visual inspection of the topographical distribution averaged across conditions. This procedure resulted in the analyses of the 40–80 Hz range for the iGBR including an electrode cluster around Pz (i.e., Cz, CPz, CP1, CP2, Pz, P1, P2, P3, P5, POz, PO3, PO4) which corresponds almost completely to the cluster chosen by Hassler and colleagues (Hassler et al. 2011). The time window from 250 to 900 ms was chosen, respectively. The time windows for both lower frequency ranges were derived from a previous study (Gruber and Müller 2002) and adapted by means of a visual inspection of the TF plot. The iABR was analyzed by means of the 10–13 Hz range including an electrode cluster surrounding POz (i.e., O1, O2, P5 P6, P7, P8, PO3, PO4, PO7, PO8) in the time window from 600 to 1000 ms. For the iTBR, the TF plot indicated two time windows of interest, with an early iTBR increase from 100 to 300 ms after stimulus onset and a later increase from 650 to 1150 ms. Both time windows were included in the analyses. An electrode cluster around Fz was chosen (i.e., Fz, F1, F2, FC1, FC2). The visual inspection of both mean topographies of the iTBR revealed an even more pronounced increase at posterior electrode sites. Given that our hypothesis is linked to the acceptance of the null hypothesis, we included this cluster into analyses to take into account that a potential effect might occur at other electrode sites than predefined by previous literature. By means of this explorative cluster, we intended to prevent false acceptance of the null hypothesis. For that reason, posterior electrodes around Pz were included as separate clusters in the iTBR analyses as well (early time window: Pz, P1, P2, P3, P4, POz, PO3, PO4; later time window: Oz, O1, O2).

### Statistical analyses

The dependend variables of this study are the response time on the behavioral level and the ERP amplitude, iGBR, iABR, and iTBR on the electrophysiological level. As outlined in the introduction, these measures are proposed to operationalize the repetition suppression effect which occurs in conventional repetition priming paradigms. We hypothesize that the response time, the ERP amplitude, and the iGBR would be lower for the second presentation compared to the first presentation. The iABR is hypothesized to be less negative in response to second presentation compared to the first presentation and it is assumed that the iTBR does not differentiate between first and second presentation. To test these hypotheses, all statistical analyses were performed using SPSS Statistics (IBM, Version 28). Normal distribution was checked by means of the Shapiro–Wilk test and by inspection of the respective Q–Q plots. According to the Shapiro–Wilk test, normal distribution can be assumed for most variables, while the Q–Q plot showed no severe violation for any variable (please see Supplementary Material 1 for details). Due to the robustness of parametric procedures with adequate sample size and the inspection of Q–Q plots, parametric procedures were applied. The response time, the ERP response, the iGBR, and the iABR to first and second presentations were analyzed by means of one-tailed paired samples *t *tests. Since the TF plot indicated two relevant time windows for the iTBR, a three-factorial rmANOVA was calculated including the factors REPETITION (first presentation, second presentation), POSITION (frontal cluster, posterior cluster), and TIME WINDOW (early time window, late time window). Regarding the resulting post hoc tests, only the comparisons of the first and second presentations are of relevance for the research question at hand, not the comparisons of the time windows and electrode clusters. Therefore, if indicated by the rmANOVA, two-tailed paired samples *t *tests were calculated only with respect to the factor REPETITION. Since our hypothesis that the iTBR does not discriminate between first and second presentation of stimuli is equivalent to accepting the null hypothesis, the alpha level for the planned comparisons was set at *α* = 0.10 and corrected for multiple comparisons by means of a Bonferroni correction (*α* = 0.10/4 = 0.025). For each test procedure, the respective effect size was calculated (partial eta squared (*η*^2^) for rmANOVA, Cohen’s *d* for post hoc *t *tests). The different lags between stimulus repetition were not included as a factor in the analysis as a preceding study found no effect of the different lags on the electrophysiological measures (Gruber et al. 2004) and moreover, does not contribute to the research question at hand.

## Results

### Behavioral data

As expected, participants responded significantly quicker to the second presentation than to the first presentation of the stimuli (*t*(28) = 11.22, *p* < 0.001; *d* = 2.05; *first presentation*: *M*_FP_ = 695.04 ms, *SD*_FP_ = 189.93 ms; *second presentation*: *M*_SP_ = 556.48 ms, *SD*_SP_ = 152.57 ms). Hence, our behavioral data verified that our paradigm was suitable to evoke the characteristics of repetition priming. Moreover, participants detected 89.19% (*SD* = 4.62%) of all stimuli depicting edible stimuli on average, i.e., approximately 90% of all stimuli presentations depicting edible objects were followed by a button press. Moreover, 94.87% of their responses were correct (*SD* = 2.95%), whereas only 5.13% were false responses (*SD* = 2.95%), i.e., 5% of all responses followed stimuli that depicted nothing edible.

### Electrophysiological data

**Fig. 3:**
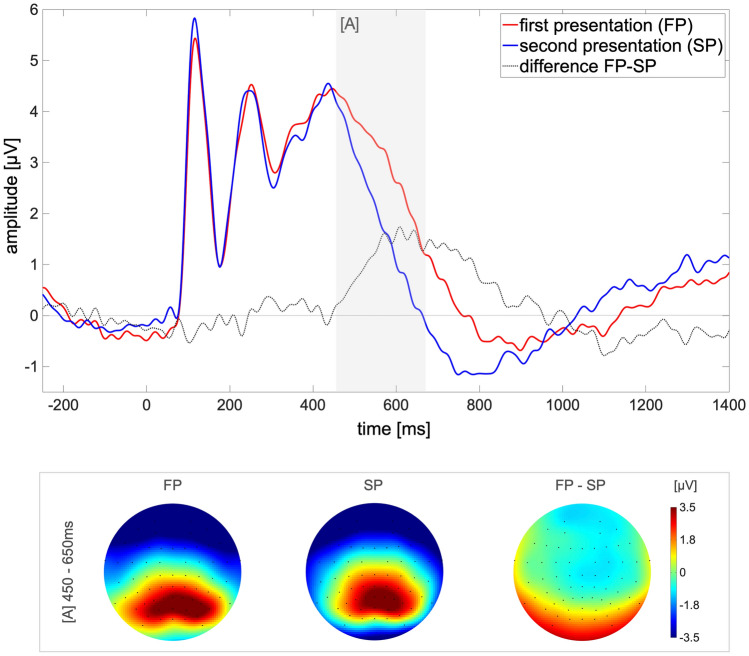
Visualization of the ERP line plot and topography as a function of the first or second presentation of the stimuli. *Note*: The line plot depicts the mean amplitude for the electrodes POz, PO3, PO4, Oz, O1, O2. The light grey, horizontal line marks the *y*-axis intersection with *y* = 0 for better visibility of the difference line deviation from zero. Please note that since the amplitudes at the most posterior electrodes are negative in value for both conditions, a negative value is subtracted from a negative value for visualization, which mathematically results in a positive value and is numerically greater than the reduction in amplitude at more central sensors included in the analysis. Hence, due to the scaling of the topographical distribution, the analyzed effect is less visible in the topography compared to the line plot of the amplitude

*Event-related potentials.* To validate that the paradigm sufficiently evoked a suppression after repeated presentation of the stimuli, the ERP was computed and compared between first and second presentation. In conformity with the hypotheses, the first presentation of the stimuli was associated with a higher amplitude of the ERP compared to the second presentation from 450 to 650 ms after stimulus onset (*t*(28)  = 4.85, *p* < 0.001, *d* = 0.90). As depicted in Fig. 3, the amplitude decreases from the first to the second presentation measured at posterior sensors. Thus, at the level of ERPs, we achieved a replication of the repetition suppression effect under VR conditions without constraints, validating the paradigm as well as the acquisition and analysis of this effect under VR conditions.

**Fig. 4:**
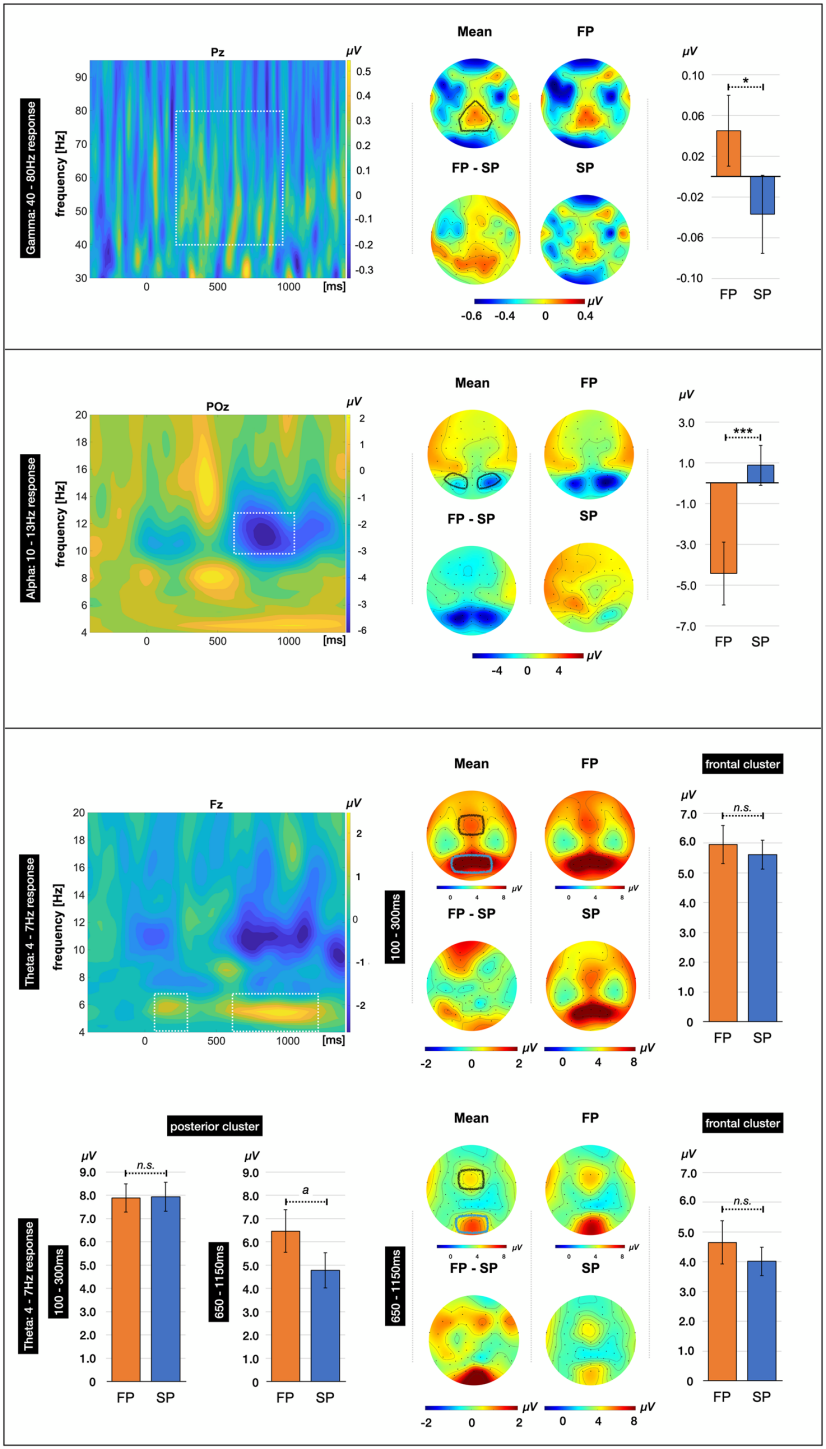
Visualization of the induced oscillatory responses in the ranges of the iGBR, iABR, and iTBR. *Note*: per frequency range, a time–frequency plot of the difference between conditions (first–second presentation) is depicted and the frequency range and time window included in the analysis are marked by a white, dotted line. The amplitude distribution is depicted for the average across conditions (mean), separately for first (FP) and second (SP) presentations, and for the difference FP *minus* SP per frequency and time window. The electrodes used for statistical comparisons are marked in the respective mean topographies. The bar plots indicate the mean amplitude for the indicated electrode cluster and time window. The error bars depict the standard error of the mean. Significant comparisons are marked, respectively, with *p < 0.05; **p < 0.01; ***p < 0.001; ^a^*p* < 0.05 but not significant after Bonferroni correction

*Induced oscillatory responses.* As hypothesized, we found a higher iGBR to the first presentation of objects compared to their second presentation (*t*(28) = 2.41, *p* = 0.012, *d* = 0.45; see Fig. 4). Regarding the iABR, the first presentation of stimuli elicited a more negative amplitude compared to the second presentation (*t*(28) = −3.90, *p* < 0.001, *d* = −0.72; see Fig. 4). The iTBR yielded significant main effects of TIMING and POSITION but not for the factor REPETITION (see Table 1). The first-order interactions did not reach significance (all *F*s (1, 28) < 3.5, all *p*s > 0.07, see Table 1); however, the second-order interaction REPETITION x POSITION x TIMING reached significance (*F*(1,28) = 4.35, *p* < 0.05, *η*^2^ = 0.13). The planned post hoc comparisons between first and second presentations of the stimuli revealed a trend effect of higher iTBR to first presentations compared to second presentations during the late time window at posterior electrode sites (*t*(28) = 2.30, *p* = 0.029, *d* = 0.43) which did not reach significance after Bonferroni correction (*α* = 0.10/4 = 0.025). The iTBR did not differentiate between first and second presentation for other combinations of time window and electrode cluster (all *t*s < 1; all *p*s > 0.35; see Table 2). Please see Fig. 4 for a visualization of the induced oscillatory responses.

**Table 1:** 2 × 2 × 2 rmANOVA for the induced thetaband response (iTBR)

	*rmANOVA*		
	*F(1,28)*	*p*	*η*^2^
Repetition	1.97	0.172	0.07
Position	14.74	< 0.001	0.35
Timing	13.43	0.001	0.32
Repetition × position	0.53	0.471	0.02
Repetition × timing	3.47	0.073	0.11
Position × timing	2.89	0.100	0.09
Repetition × position × timing	4.35	0.046	0.13

**Table 2:** Planned two-sided post hoc *t *tests for the induced thetaband response (iTBR) to first presentations (FP) and second presentations (SP)

	*Two-tailed t test for paired samples*		
	*t*(28)	*p*	*|d|*
*FP-SP*			
Frontal iTBR 100–300 ms	0.65	0.524	0.12
Frontal iTBR 650–1500 ms	0.92	0.366	0.17
Posterior iTBR 100–300 ms	−0.10	0.922	0.02
Posterior iTBR 650–1500 ms	2.30	0.029	0.43

## Discussion

### Summary

The objective of the study at hand was twofold: first, it aimed to examine the feasibility of investigating induced oscillatory responses under VR conditions and second, whether the modulations of both, the low- and the high-frequency range, occur equivalently to conventional EEG paradigms when translated to virtual reality (VR) conditions by means of the replication of a well-documented repetition suppression effect related to implicit memory. The latter aimed to provide further insights into mnemonic processing under VR conditions. The repetition priming paradigm offers a robust, widely replicated, and well-documented starting point to examine the electrophysiological level under conditions differing from standard settings. In the course of the following discussion, we will elaborate on the degree to which our findings can be assimilated into the prevailing research framework. We aim to discern the extent to which our results align with prior research albeit using a less frequently applied VR–EEG approach, i.e., the utilization of a head-mounted display during the EEG data acquisition intended for analysis at high-frequency levels. Through this synthesis, we aim to derive consequences and outlooks for future VR–EEG studies.

Our results provide a next step to unravel differences found between VR-based paradigms and conventional paradigms indicating modulations of the cognitive mechanisms and processes underlying these different modalities. To check whether the paradigm per se elicited priming effects, the response times and ERP complex were inspected before considering the oscillatory responses. As expected, participants responded faster to the repeated presentation of stimuli compared to their first presentation. In line with this, the ERP measured at posterior sensors exhibited a lower amplitude in response to repeated presentations. The examination of the oscillatory responses drew a congruent picture: as hypothesized, first presentations of stimuli exhibited a higher induced gammaband response (iGBR) and a more negative induced alphaband response (iABR) compared to second presentations. The induced thetaband response (iTBR) considered at frontal electrodes did not differentiate between first and repeated presentations. The visual inspection of the respective topographical amplitude distributions indicated a posterior effect with a higher iTBR for first presentations which might be related to explicit encoding mechanisms. This effect, however, only reached trend-level after Bonferroni correction.

### Priming and repetition suppression of event-related responses

Indicating successful priming, participants responded about 140 ms faster to repeated presentation of stimuli compared to the first presentation. Congruently, the amplitude of the ERP was lower from 450 ms post-stimulus for repeated presentations, corresponding to a later component of the ERP labeled L2 in previous studies (see e.g., Gruber et al. 2004; Gruber and Müller 2002) which has been associated with faster and more efficient processing of objects due to their repeated presentation (Guo et al. 2007). As the first presentation was followed by a lag of none, one or four objects before being presented a second time, habituation can be excluded as an alternative explanation of the speed gain and reduced amplitude (Gruber et al. 2004; Gruber and Müller 2002). Moreover, the repetition suppression effect is proposed to attenuate when a greater lag occurs between the first and second presentation (e.g., Gruber and Müller 2002; Guo et al. 2007). Averaging over the three different lags could, thus, potentially diminish the effect. Nevertheless, we observed a significantly reduced amplitude for repeated presentations with high effect size (*d* = 0.9), which is indicative of the robustness of the effect (see also Johnsdorf et al. 2023) and substantiates the potential of this paradigm to investigate further neural domains. Likewise, reduced attention (Guo et al. 2007) can be excluded as an explanation of the observed effect, since our participants had to identify a certain object category in random trials. With a response accuracy of approximately 95%, it can be assumed that there was no relevant attentional drop that would exclusively affect the second presentations. The frequent explanation that repeated presentation of objects leads to the sharpening of the neural representation of the object, and thus to reduced responses to repeated presentations, is also coherent for our data (e.g., Grill-Spector et al. 2006; Gruber et al. 2004b; Snyder and Keil 2008). Yet the ERP complex might particularly reflect the involvement of brain systems sensitive to task-irrelevant, incidental stimulus repetition (e.g., Rugg et al. 1995) and is, thus, thought to play a functionally different role in visual perceptual processes as oscillatory responses (Gruber et al. 2004).

### Modulations of high-frequency oscillations reflect neural sharpening

Regarding the frequency domain, the joint VR–EEG setup allowed for a successful replication of the frequently reported repetition suppression effect by means of a significantly lower iGBR to second compared to first presentations. As outlined in the introduction, the iGBR is associated with the neural object representation including both perceptual and semantic features of the respective objects. The reduced iGBR in response to repeated presentations is proposed to mirror neural sharpening of said object representation which is predominantly associated with the sharpening of semantic features (Friese et al. 2012a, b).

Albeit we found said higher iGBR to first compared to second presentations, the amplitude of both iGBRs was relatively small compared to the majority of previous studies following a comparable procedure (e.g., Hassler et al. 2011), with the iGBR to second presentations even yielding negative values in our study. However, small amplitudes have previously been reported in similar settings as well (Graetz et al. 2019). It is unlikely that the frequency responses are generally underestimated under VR conditions. Although other studies found differences in the magnitude of various frequency responses when immersive VR and 2D screens are compared (Bilgin et al. 2019; Dan and Reiner 2017; Kisker et al. 2021; Schöne et al. 2023), the magnitude of frequency responses in VR is in some cases also higher than in response to screen-based paradigms (Bilgin et al. 2019; Xu and Sui 2021). In particular, Bilgin and colleagues (Bilgin et al. 2019) found no difference in the lower iGBR (30–45 Hz) in response to emotional environments presented either via an head-mounted display or a 2D screen. These results suggest that the differences in magnitude depend on the paradigm as well and not solely on the use of VR-head-mounted displays.

Since the iGBR to the stimulus was preceded by a fixation used as baseline, a possible explanation for the relatively small amplitudes might be that there is already a relatively high iGBR during baseline. As Busch and colleagues (Busch et al. 2004) pointed out, the offset of a stimulus potentially yields a burst in the iGBR just like the stimulus onset. Consequently, it is recommended to choose a presentation time of the stimulus longer than the time window to be analyzed to avoid an overlap of offsets and subsequent onsets (Busch et al. 2004). With a presentation duration of 2000 ms and a randomized intertrial interval of 1500–2500 ms, we are optimistic that the responses to the current and to the previous trial do not overlap. However, unlike in conventional studies, the cube used for fixation might be perceived as a consistent, 3D object and therefore might have yielded a relatively high iGBR during baseline, which would make the actual synchronization of interest during stimulus presentation seem smaller in comparison. So far, however, there is little insight into the effects of switching from two-dimensional to three-dimensional stimuli, in particular concerning the fixation. To the best of our knowledge, no study has yet examined the iGBR beyond 50 Hz to 2D and 3D stimuli, especially not in immersive VR settings. Alternatively, the mean amplitude might appear relatively small since the iGBR can also occur in bursts rather than in a strong sustained response, as shown in Fig. 4. When averaged, such bursts would result in a smaller average amplitude compared to a sustained response. Such bursts have been associated with theta-gamma coupling: as reported in previous studies on mnemonic processes, the gammaband response can in cases be nested into the thetaband oscillations in memory paradigms, and is proposed to reflect top-down control on the recall of neural representations, i.e., by means of attentional sampling, mnemonic updating and predictive coding (e.g., Köster and Gruber 2022; Graetz et al. 2019, Friese et al. 2013). However, analyses to examine the phase-amplitude coupling of the two frequency bands was beyond the scope of our study and, consequently, our data do not allow conclusions about this assumption.

Another possible explanation for the relatively small amplitudes is that fewer cognitive resources might be needed to process the stimuli under VR conditions, since, for example, depth information is immediately available from the 3D environment (see Schöne et al. 2021a, b). A previous study found that processing 3D stimuli yields lower cognitive load compared to the same material presented on a 2D screen (Dan and Reiner 2017) and in similar vein, lower recall effort was associated with VR-based encoding compared to screen-based encoding (Kisker et al. 2021). As the iGBR is associated with the integration of perceptual features (e.g., Köster and Gruber 2022), it might be lower if this bottom-up integration process is facilitated, for example, by immediately available depth stimuli. Other findings imply a higher need for resources in VR, especially during encoding processes (Slobounov et al. 2015). Regarding these heterogeneous results, this explanation to date remains a conjecture that goes beyond the research question at hand and requires further investigation. Nevertheless, we replicated the hypothesized repetition suppression effect, suggesting that modulations in the high-frequency range are also detectable under VR conditions and modulated by underlying cognitive mechanisms and processes in line with previous findings.

### Top-down inhibitory processes are mirrored in lower frequency spectral changes

Complementing the high-frequency domain and as hypothesized, the iABR was more negative for initial presentations compared to repeated presentations, indicating lower cortical activity during the latter (Davidson et al. 2000; Harmon-Jones et al. 2010; Klimesch 1999; Neuper and Pfurtscheller 2001). In line with the neural sharpening of object representations, inhibitory processes as reflected by alphaband desynchronization (Fries et al. 2008a, b; Klimesch et al. 1997) might be stronger during initial presentations due to the stimuli's novelty and thus, during the initiation of the formation of a neural object representation. Vice versa, a less negative iABR to repeated presentations might result from top-down suppressive mechanisms, i.e., the suppression of irrelevant information (see Jensen and Mazaheri 2010; Sauseng et al. 2009). Hence, the reduced magnitude of the iABR’s negativity as a marker for visual processing load (see e.g., Sauseng et al. 2009; Fries et al. 2008a, b; Klimesch et al. 1997) might be the low-frequency counterpart to the sharpening mechanism reflected in the iGBR. Interestingly, previous studies implementing repetition priming paradigms did not reveal a similar effect in the iABR (Gruber et al. 2004). Albeit the alpha power decreased in response to the stimulus presentation per se, it did not differentiate between initial and repeated presentations (Gruber et al. 2004). Other studies found the reversed effect, i.e., a more negative iABR to repeated presentations (Gruber and Müller 2002). However, these studies analyzed relatively early time windows after stimulus onset, while our TF plot indicated a later time window of interest.

Proceeded by a relatively early onset of the iGBR’s modulation, the iABR yielded relatively late effects, setting in later than 500 ms after stimulus onset. In synthesis with the participants’ behavioral response, which decreased from about 700 ms for first presentations to about 550 ms for second presentations, the results implicate the involvement of top-down processes. For example, the desynchronization of the iABR is associated with visual matching of previously encountered stimuli with the mental representation, while a reduction of this desynchronization reflects less cortical activity, and thus lower attentional demands and visual load to match stimulus and representation (Kisker et al. 2021; Sauseng et al. 2009). This might be facilitated by priming effects like repetition suppression of the iGBR and hence, by neural sharpening. In a similar vein, Johnsdorf and colleagues (Johnsdorf et al. 2023) argued that the initial processing of a stimulus under VR conditions is more complex and demanding compared to conventional 2D settings. However, this extensive process might lead to more optimal and efficient processing in the encoding process (Johnsdorf et al. 2023), which, in our study, might be mirrored in the modulation of both, the earlier effect in the high-frequency range as well as the later effect in the low-frequency range. Future studies might further clarify the degree to which different oscillations are influenced by top-down task requirements through the examination of the task-related significance of the repetition, e.g., by means of distinct responses to initial and subsequent presentations. A previous study revealed the suppression of the iGBR in response to repeated stimuli if the task demands are the same for the first and repeated presentation of this stimuli, while the modulation ERP response was independent of the task (Gruber et al. 2006). It remains an open question whether these results would equally transfer to VR conditions.

In contrast to the iGBR and the iABR, but as expected, the frontomedial iTBR did not differ as a function of presentation frequency. This finding corresponded to our hypothesis since the iTBR is primarily associated with explicit memory processes. However, a late, posterior iTBR effect reached trend level with a higher amplitude for initial compared to repeated presentations. Due to the iTBR’s association with memory formation, it might be indicative of an object becoming familiar due to repeated presentations (Lafontaine et al. 2016) in which priming may serve as an encoding mechanism that initiates the formation of memory traces (see Gotts et al. 2012). An alternative explanatory approach for the observed trend effect might be the thetaband’s modulation depending on depth cues (Tang et al. 2022). Presenting the same objects either as graphical representations on a 2D screen or in a stereoscopic manner via a VR head-mounted display, Tang and colleagues (Tang et al. 2022) found a stronger iTBR to the latter which was associated with the matching of the perceived 3D shape and its neural visuo-spatial representation. While the latter interpretation is in line with the iTBR being a marker of memory formation, the depth information was not varied between first and second presentations in our study, and hence it is unlikely that the thetaband response was modulated by depth perception in this paradigm. Nevertheless, the observed trend effect could be an interesting starting point for further studies.

In summary, we replicated previous findings in terms of repetition suppression at the behavioral level, the ERP level, and most importantly, in the frequency domain using a joint VR–EEG repetition priming paradigm. These results are in congruence with previous studies and support the robustness and transferability of the findings on neural sharpening of object representations on the one hand, and the feasibility of joint VR–EEG settings even for the high-frequency domain on the other hand. Even more, we found later modulations of the iABR and a comparable trend in the iTBR, which might involve the onset of top-down processes, and might thus mark the formation of a memory trace instantiated by priming (e.g., Gotts et al. 2012).

### Implications for joint VR–EEG experiments

Our results demonstrate that VR as an experimental setting contributes not only to transferring previous findings to more sensory-rich settings, but also to extending them. In our study, the acquisition and analysis of the iGBR was proposed to be most difficult to realize in a joint VR–EEG study as the high-frequency ranges are even more prone to electrical interference, and potential electrical noise from the VR device coincides with the frequency range of interest (Weber et al. 2021; see also Introduction). To check the data for electrical interference, an FFT was calculated for each individual data set as a first step. Participants with peaks in the 50 Hz range and its harmonics, i.e., 100 Hz, were excluded. Such peaks were only found in one data set. As described in the method section, participants were seated in an electrically shielded cabin (faraday cage). All electrical devices were placed outside this cabin beside the EEG system, the HTC Vive Pro2 head-mounted display, and two base stations for head-tracking, while placing connectors and transmitters of the latter outside the cabin as well. The only difference between the disrupted dataset’s acquisition and all further datasets was that a power bank was left within the electrically shielded cabin during the data acquisition of this participant (see Electrophysiological recordings and preprocessing). The interference in this dataset by a basic power bank demonstrates in all clarity that not every setup is suitable and must be meticulously controlled if the high-frequency range is to be analyzed. The HTC Vive Pro 2 was used similarly with all participants and its use did not generally result in electrical interference in the 50 Hz range. Albeit the solution to shield the EEG system from further electrical interference seems arbitrary and is common standard in studies examining high-frequency oscillations (e.g., Busch et al. 2004, 2006), previous studies aiming for the analyses of the high-frequency range (e.g., Kim et al. 2001; Tarrant and Cope 2018; Wang et al. 2020) or examining the quality of joint VR–EEG applications did not state whether they did so (e.g., Weber et al. 2021). As a result, previous studies have not distinguished whether noise in the 50 Hz range originates from the head-mounted display per se, or whether other power sources, e.g., VR systems power connectors, are critical for line hum. Even though we did not vary whether the data were measured inside or outside the cabin, the one data set we had to exclude due to the power bank demonstrates that electrical shielding was essential for our signal quality.

However, as previous studies have demonstrated that the specific combination of VR and EEG systems is critical (Weber et al. 2021), the piloting of any combinations of the specific EEG system and the specific VR device, respectively, is essential and should precede any study aiming at frequencies around and beyond 50 Hz. In this context, our study provides an overarching insight that under control of known interfering factors, e.g., electrical interference (e.g., Weber et al. 2021) or stimulus size (Busch et al. 2004), it is feasible to include the high-frequency range under VR conditions, which is indispensable, for example, for a deeper understanding of mnemonic processes. In this line of thought, the iGBR and iTBR are not only associated with implicit and explicit memory processes, respectively (e.g., Gotts et al. 2012), but also allow for a differentiation of familiar and recollection-based memories in episodic memory (e.g., Gruber et al. 2008). Notably, this specific differentiation was not implemented on the electrophysiological level in previous VR-based approaches to episodic memory (Kisker et al. 2021), which left questions about the differences between memory processes resulting from settings of varying immersion not exhaustively answered. Consequently, for future research approaches, the interaction of different markers might play a crucial role to unravel the processes and mechanisms occurring in immersive environments and underlying these experiences, while controlling for confounds like external electrical interference**.**

## Conclusion

As outlined in the discussion’s summary, and most importantly, we replicated the well-documented repetition suppression effect on the behavioral level, the ERP level, i.e., priming, and in both the low-frequency and high-frequency domain under VR conditions. In accordance with the hypotheses, participants responded faster to repeated compared to first presentations of stimuli, the iABR exhibited less negative values, and the iGBR decreased while the iTBR did not differentiate between first and second presentations of stimuli. Notably, the analysis of the iGBR was largely omitted or only limitedly accessible in previous joint VR–EEG studies. To obtain these modulations of the iGBR congruent with previous research, we electrically shielded the EEG acquisition using a Faraday cage. Only one dataset needed to be excluded due to line hum which was most likely not caused by the VR head-mounted display, which was equally used by all participants. Consequently, the study illustrates the possibility to investigate induced frequency oscillations even above 50 Hz and the need to pilot the specific combination of EEG and VR systems, but likewise the opportunity to further unravel key mechanisms in cognitive functions like mnemonic processing as reflected in the oscillatory responses under VR conditions.

### Supplementary Information

Below is the link to the electronic supplementary material.

Supplementary file1 (PDF 134 KB)

## Data Availability

The datasets generated during and analyzed during the current study are available from the corresponding author on reasonable request.

## Abbreviations

EEG: Electroencephalography
ERP: Event-related potential
FFT: Fast Fourier transformation
iABR: Induced alphaband response
iGBR: Induced gammaband response
iTBR: Induced thetaband response
VR: Virtual reality
