# Supplementary material for: Induced oscillatory brain responses under virtual reality conditions in the context of repetition priming
Source: Exp Brain Res. 2024 Jan 10;242(3):525–41. doi: 10.1007/s00221-023-06766-8 (PMC10894769; doi:10.1007/s00221-023-06766-8)
Supplement: Supplementary file 1 — Supplementary file1 (PDF 134 KB) [file 221_2023_6766_MOESM1_ESM.pdf]

Supplementary material: *Induced Oscillatory Brain Responses under Virtual Reality Conditions in the Context of Repetition Priming.*

Table S1. Shapiro-Wilk-test for normal distribution for all variables included in the analyzes.

	Shapiro-Wilk-test		
	statistic	*df*	*p*
FP_iGBR_250_900	0.964	29	0.408
SP_iGBR_250_900	0.949	29	0.177
FP_iABR_600_1000	0.822	29	<.001
SP_iABR_600_1000	0.974	29	0.683
FP_frontal_iTBR_100_300	0.881	29	0.004
SP_frontal_iTBR_100_300	0.942	29	0.112
FP_frontal_iTBR_650_1150	0.916	29	0.024
SP_frontal_iTBR_650_1150	0.974	29	0.682
FP_posterior_iTBR_100_300	0.956	29	0.259
SP_posterior_iTBR_100_300	0.91	29	0.018
FP_posterior_iTBR_650_1150	0.93	29	0.056
SP_posterior_iTBR_650_1150	0.969	29	0.52
FP_RT	0.927	30	0.04
SP_RT	0.948	30	0.145
FP_ERP	0.984	29	0.933
SP_ERP	0.974	29	0.683

Note. Significant results contradicting the assumption of normal distribution were highlighted in grey. Additionally, the Q-Q-plots were checked, which did not indicate severe violation of normal distribution. Due to the robustness of parametric procedures with adequate sample size and inspection of Q-Q plots, parametric procedures were retained.

Abbreviations

FP – first presentation,

SP – second presentation

iGBR – induced gamma band response

iABR – induced alpha band response

iTBR – induced theta band response

RT – response time

ERP – event related potential
